# Towards a Connected Mobile Cataract Screening System: A Future Approach

**DOI:** 10.3390/jimaging8020041

**Published:** 2022-02-10

**Authors:** Wan Mimi Diyana Wan Zaki, Haliza Abdul Mutalib, Laily Azyan Ramlan, Aini Hussain, Aouache Mustapha

**Affiliations:** 1 Department of Electrical, Electronic and Systems Engineering, Faculty of Engineering and Built Environment, Universiti Kebangsaan Malaysia (UKM), Bangi 43600, Malaysia; wmdiyana@ukm.edu.my (W.M.D.W.Z.); p102323@siswa.ukm.edu.my (L.A.R.); draini@ukm.edu.my (A.H.); 2 Optometry and Vision Science Programme, Faculty of Health Sciences, Universiti Kebangsaan Malaysia, Jalan Raja Muda Abdul Aziz, Kuala Lumpur 50300, Malaysia; 3 Division Telecom, Center for Development of Advanced Technologies (CDTA), Baba Hassen, Algiers 16081, Algeria; maouache@cdta.dz

**Keywords:** cataract, image processing, imaging modalities, artificial intelligence (AI)

## Abstract

Advances in computing and AI technology have promoted the development of connected health systems, indirectly influencing approaches to cataract treatment. In addition, thanks to the development of methods for cataract detection and grading using different imaging modalities, ophthalmologists can make diagnoses with significant objectivity. This paper aims to review the development and limitations of published methods for cataract detection and grading using different imaging modalities. Over the years, the proposed methods have shown significant improvement and reasonable effort towards automated cataract detection and grading systems that utilise various imaging modalities, such as optical coherence tomography (OCT), fundus, and slit-lamp images. However, more robust and fully automated cataract detection and grading systems are still needed. In addition, imaging modalities such as fundus, slit-lamps, and OCT images require medical equipment that is expensive and not portable. Therefore, the use of digital images from a smartphone as the future of cataract screening tools could be a practical and helpful solution for ophthalmologists, especially in rural areas with limited healthcare facilities.

## 1. Introduction

Ocular diseases affecting the anterior segment of the eye are the leading cause of ocular morbidity. These include dry eye conditions, infections, traumas of various types, inflammatory reactions, hereditary disorders, and cataracts. Individuals with these disorders may experience continual progression and deterioration of symptoms, which can result in varying degrees of vision loss with or without pain [1]. Cataracts are anterior segment ocular illnesses characterized by a decrease in lens transparency owing to lens opacification, which can result in vision impairment or blindness. According to the systematic review and meta-analysis by Flaxman et al. [2], cataracts are one of the leading causes of moderate or severe vision impairment in the global population, with a total of 52.6 million people affected in 2015. They are also one of the leading causes of blindness affecting a total of 12.6 million people in 2015. Furthermore, by 2020, it is projected that the number of people affected by cataract-related vision impairment and blindness will rise. WHO stated that near or far vision impairment affects at least 2.2 billion people globally. However, vision impairment could have been prevented or addressed in at least 1 billion—or nearly half—of these cases. 94 million people out of those billion people had moderate or severe distance vision impairment or blindness due to cataract [3]. In addition, cataracts were also one of the most common causes of low vision in Malaysia under the ‘various types of crystalline lens disease’ category [4]. Globally, cataracts are the prominent cause of blindness among low- and middle-income countries, e.g., Malaysia, China, and Pakistan. A shortage of ophthalmologists is an urgent problem, especially in rural areas [5,6,7].

**Figure 1:**
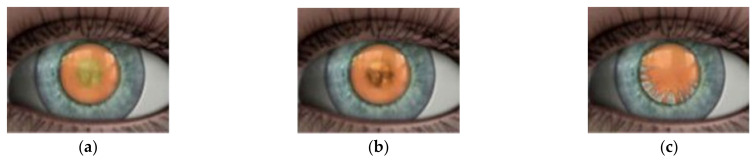
(**a**) Nuclear Cataract, (**b**) Cortical Cataract, (**c**) Posterior Capsular Cataract [9].

Cataracts are classified into three types: age-related cataracts, paediatric cataracts, and cataracts secondary to other causes. Age-related cataracts are the most common type in adults, typically developing between the ages of 45 and 50 [8]. Age-related cataracts are classified into three types according to the location of the opacification within the lens: nuclear cataract, cortical cataract, and posterior subcapsular cataract (PSC) (refer to Figure 1 [9]). A nuclear cataract is a clouding of the lens located in the lens’s centre and it is also the most common type of cataract associated with advanced age [10]. A cortical cataract is typically wedge-shaped, beginning at the cortex and extending to the centre of the lens. A plaque-like opacity develops in the axial posterior cortical layer in posterior subcapsular cataracts. In most cases, an individual will be diagnosed with more than one type of cataract.

Currently, computer-aided diagnostics (CAD), a concept that combines the skills of physicians and computers, has established itself as a prominent area of research in medical imaging and diagnostic radiology. Automated computer analysis, a well-established research topic in medical imaging, is an approach that is entirely reliant on computer algorithms. The automated detection technique usually consists of image pre-processing, feature extraction, feature selection, segmentation, and classification [11]. Significant advances in computing and artificial intelligence (AI) technology, such as machine learning (ML) and deep learning (DL), as well as big data analytics, enable radiologists and ophthalmologists to gain a level of clinical decision support that significantly reduces diagnostic errors. Other than being the most widely used artificial intelligence (AI) method for a variety of tasks including medical imaging, DL has also been shown to be successful at detecting clinically significant features for the diagnosis and prognostic prediction of ocular diseases [12,13]. One could argue that computer-assisted diagnostics shorten diagnostic time, expedite disease examination, and aid in locating affected areas [14].

Imaging modalities play a critical role in routine ophthalmologic practice. It is nearly impossible to conduct ophthalmic examination without employing appropriate imaging modalities [15]. Ophthalmic imaging modalities, including slit-lamp images, fundus images, and OCT images have been widely used for cataract detection and grading. The slit-lamp camera is high-intensity light source equipment that consists of two components: a corneal microscope and a slit-lamp. The slit-lamp image is captured with a slit-lamp camera, which is typically used to inspect the anterior and posterior segments of the human eye. In a typical clinical setting, an ophthalmologist would grade cataracts based on slit-lamp images by comparing them with standard grading protocols, such as LOCS III or Wisconsin. The fundus images are captured with the fundus camera, which is a specialized low-power microscope with a connected camera that can view the internal surface of the retina, blood vessels, posterior pole, optic disc (OD), and macula [16]. Ophthalmologists will use the images to diagnose and treat eye diseases such as diabetic retinopathy (DR), glaucoma, cataracts, age-related macular degeneration, and retinal detachment. OCT is a type of imaging modality that generates two- and three-dimensional cross-sectional images of tissue by combining numerous axial scans into a composite B-scan. Previously, the anterior segment (AS) was evaluated mostly with ultrasound biomicroscopy. However, ultrasound biomicroscopy acquires images at a significantly slower rate than AS-OCT, at eight frames per second versus 4000 frames per second for the latter [17]. With the development of AS-OCT, it is now possible to perform a more thorough examination of the anterior chamber.

Some of the medical equipment needed for cataract detection or screening by an experienced ophthalmologist is costly, and current manual methods are time-consuming, subjective, and dependent on ophthalmologists’ experience. The ophthalmoscope and other imaging equipment used in the diagnosis of eye problems require highly skilled and trained doctors. In Malaysia, the current optometrist-to-patient ratio is believed to be approximately 1 to 22,000. This is concerning, as the World Council of Optometry (WCO) recommends a ratio of one to ten thousand [18]. This figure also demonstrates that we continue to have a shortage of practicing optometrists, particularly in rural areas. To address this issue, researchers have developed several methodologies that enable automated cataract identification and grading employing a variety of ophthalmologic imaging modalities, including fundus images, slit-lamp images, optical coherence tomography (OCT) images, and digital images. The advancement of methods and techniques for cataract detection and grading has resulted in the development of CAD or automated computer analysis, which has aided ophthalmologists significantly, particularly in rural areas with limited access to quality healthcare facilities. The purpose of this article is to provide an overview of the approaches and techniques developed over the last few years for cataract identification and grading. This article will first review the traditional clinical cataract assessment in Section 2. In Section 3, the discussion will expand on past works on methodologies and strategies for automated cataract diagnosis and grading using different approaches, including image processing, machine learning, deep learning, and other available tools for cataract grading. Next, Section 4 will discuss the modern trends in cataract screening and Section 5 will discuss the challenges and future direction. Lastly, Section 6 will be the conclusion.

## 2. Traditional Clinical Cataract Assessment

Objective qualitative and quantitative evaluation of the lens is critical for any epidemiological or therapeutic investigation of cataracts, as well as for understanding the natural history of different cataract forms. Typically, several methods are used to evaluate the status of cataracts since no single available method is adequate for cataract evaluation. Previously, the available methods for cataract evaluation included clinical cataract classification and grading, resolution test target projection ophthalmoscopy, photography and other forms of image capture, ultrasound, light-scattering analysis, and fluometry [19].

### 2.1. Manual Methods for Cataract Assessment

Currently, cataract detection and diagnosis are conducted through a series of tests, including visual acuity testing, dilated eye examinations, retinal examinations, and slit-lamp examinations. Visual acuity tests are performed with the aid of a chart that measures how well a person sees at various distances. This is the most commonly used method for calculating the impact of cataracts [20]. A dilated eye exam is a common diagnostic procedure used by optometrists and ophthalmologists to better examine the interior of the eye. It expands the field of view, allowing the doctor to see more of the inside of the eye. A special device called an ophthalmoscope is used in a retinal exam to examine the back of the person’s eyes (retina) for signs of cataract. Under magnification, a slit-lamp examination allows the ophthalmologist to see the structures at the front of the person’s eye. The slit-lamp is a bright line of light (a slit) that illuminates the cornea, the iris, the lens, and the space between the iris and the cornea. In addition, the patient should be evaluated for best-corrected visual acuity, refraction, and contrast sensitivity; intraocular pressure; and examination of the patient’s other anterior segment structures, including the iris and the cornea, for possible retinal lesions that could impair final visual acuity following surgery.

**Figure 2:**
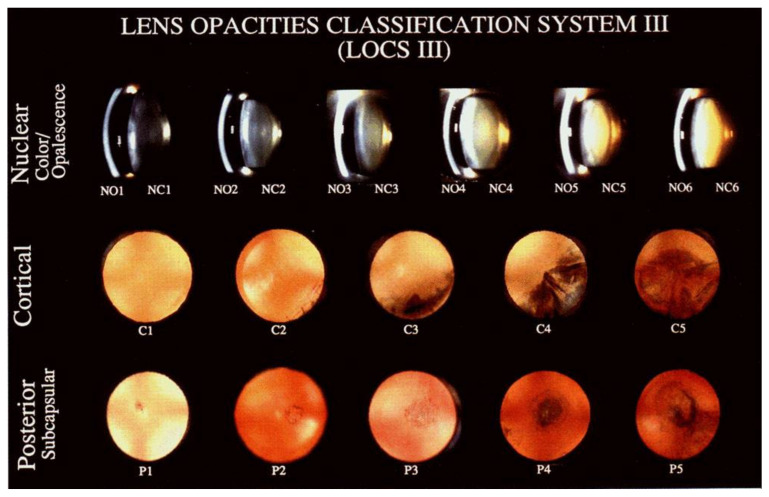
LOCS III Grading Standard [23].

Following these tests, ophthalmologists usually perform cataract grading, which involves assessing the degree of opacification to determine the severity of the cataracts. Clinical grading, such as Lens Opacities Classification [21], is commonly performed by comparing the patient’s images observed through the camera with a set of reference photographs. Several classification systems, including the Oxford Clinical Cataract Classification and Grading System and the Lens Opacities Classification System III (LOCS III), are particularly advantageous for epidemiologic investigations, anti-cataract medication trials, and clinical trials in which cataract hardness is an important factor. The Lens Opacities Classification System III (LOCS III) and the Wisconsin Grading System (WGS) are the conventional grading systems most extensively used by ophthalmologists to classify cataracts [22]. The LOCS III system [23] uses six slit-lamp images for grading nuclear colour and nuclear opalescence and five retro illumination images for evaluating cortical cataract and posterior subcapsular cataract, as illustrated in Figure 2. It evolved from LOCS II, which had the following shortcomings: (1) the nuclear colour scale was small and coarse, (2) the early stages of nuclear cataracts and posterior subcapsular cataracts were underrepresented, (3) the scaling intervals for all types of cataracts were unequal, and (4) integer grading of features was not sensitive enough to detect small changes in cataracts [19]. The standard images used in this system depict the borders of the scaling intervals, with higher scores indicating more advanced opacity. Clinical grading systems are subjective, resulting in inconsistencies over time and between observers.

### 2.2. The Importance of An Early Cataract Detection

The removal of cataracts might be necessary under several circumstances. They include facilitating ocular fundus visualization (for glaucoma monitoring or in preparation for photocoagulation therapy in diabetic retinopathy), removing a foreign body embedded in the lens, preparing for vitrectomy and surgical repair of retinal detachment, and a variety of pathologic conditions in which the lens is threatening the eye’s viability. The need for surgical intervention is determined by the patient’s needs, his or her desired level of activity and recreation, the symmetry of the disease process, the condition of other ocular structures, the patient’s general health, and appropriate informed consent with reasonable expectations [24]. In the developed world, surgery is usually considered when the anticipated improvement in vision relative to current conditions justifies the risk of major, sight-threatening complications. Previously, cataracts were not treated by surgery until they were well advanced due to relatively rudimentary surgical procedures and inadequate visual rehabilitation (no lens implants). Surgery is now performed at a much earlier stage since procedures have become more sophisticated and safer with improved visual results. If cataracts have progressed to an advanced degree, the chances of significant complications will increase. In contrast, cataract blindness is a considerably larger problem in the developing world because most people do not seek help until their cataract has progressed or lens-induced glaucoma has caused a painful loss of vision in one eye. Causes for this delay include a lack of awareness about cataract treatment, sex bias, low socioeconomic conditions, and the absence of government-sponsored welfare programs for seniors. Many countries lack enough clinicians to meet demand, and most existing doctors prefer to work in larger cities due to poor infrastructure, education, and civic amenities in rural areas. As a result, there is a huge discrepancy in how eye care is distributed [25].

## 3. Automated Cataract Detection and Grading

Ophthalmic imaging has advanced from simple photographic documentation of the condition to a robust and more progressive investigation method. This allows the ophthalmologist to make objective measurements and assessments of the detailed ocular structures that were previously unavailable in a traditional clinical examination using ophthalmoscopy. Advances in imaging techniques have resulted in a more complete understanding of the eye in health and disease. They also help identify previously undiagnosed conditions, provide a more detailed description of disease phenotypes, and serve as an objective tool for evaluating treatment efficacy and safety [26]. Traditionally, cataract was diagnosed with several tests, including visual acuity testing, dilated eye examinations, retinal examinations, and slit-lamp examinations. Cataract grading involves comparing slit-lamp images to a set of standard photographs determined by a grading protocol such as LOCS III or WGS. Recent advancements in CAD techniques, which are defined as the subset of artificial intelligence (AI), are becoming more apparent in ophthalmology [27]. These advances have prompted other researchers to investigate alternative imaging modalities, including OCT and fundus images, for use in cataract grading. This resulted in the development of new cataract grading techniques or methods that utilise image processing, machine learning, and deep learning and incorporate a variety of imaging modalities, including OCT images, fundus images, and slit-lamp images, which will ultimately enable automated cataract detection and grading.

In general, cataract diagnosis typically starts with a slit-lamp examination and is followed by physician analysis based on the slit-lamp image. The classification will be done based on the doctor’s evaluation of the turbid area of the pupil [28]. The presence and severity of the cataract will be graded by comparing its appearance in slit-lamp images in contrast with a set of standard reference photographs. The reference is normally based on grading protocols such as LOCS III and Wisconsin Grading System (WGS) which usually results in a subjective interpretation. Fundus images utilise a fundus camera to capture colored pictures of the inside of the eye to record the event of scatters and monitor their changes after some time. A retinal fundus camera is a specialized low power microscope camera connected to snap the interior part of the eye including the optic disc (OD), fovea, macula, retina, retinal veins, and back post. These retinal images are used by ophthalmologists to help identify, diagnose, and treat eye infections including DR, glaucoma, cataract, age-related macular degeneration, and retinal detachment. OCT imaging uses near-infrared light to measure the optical reflectivity profile of the tissue. This is a painless, non-invasive imaging modality that creates three-dimensional images of the retina in a matter of seconds [26]. There are several applications that utilise OCT before and after the cataract surgery including anterior lens capsule and lens epithelium evaluation in senile cataract and Fuchs’ heterochromic cyclitis using spectral-domain anterior segment OCT (SD-OCT), investigation of clear corneal incision in manual phacoemulsification and femtosecond laser-assisted cataract surgery using SD-OCT, capsular block syndrome evaluation before and after treatment using SD-OCT, and IOL power calculation (true net power measurement) in post-myopic excimer laser eyes using SD-OCT [29]. For further knowledge of the cataract diagnosis based on digital imaging, the interested researchers are recommended to refer to [30,31,32].

### 3.1. Cataract Detection with Machine Learning and Image Processing

Currently, machine learning and image processing are widely used by researchers in their studies to develop cataract detection methods. Several imaging modalities, including OCT images, fundus images, slit-lamp images, and digital images, are employed; among these, fundus and slit-lamp images are the most frequently used for cataract detection and grading. For example, Yang et al. [33] proposed a neural network classifier to automatically classify cataracts using fundus images. The proposed method divides cataract severity into four categories based on the degree of clarity of the fundus image (normal, mild, medium, or severe). As part of pre-processing, they used an improved version of the top-bottom hat transformation, which allows them to see the blood vessels in the fundus image more clearly. As a classifier, they used a 2-layer backpropagation (BP) neural network. They were able to achieve true positive rates of 82.1% and 82.9% in training and test, respectively, a promising result for early work on the automated cataract classification method. However, the pre-processing step takes longer for a single image. This is something that needs to be improved in the future. In a different approach, Behera et al. [34] proposed an automated model for cataract detection using image processing and machine learning techniques. The pre-processing step involves image processing techniques, including resizing, and smoothing, histogram equalization (CLAHE), and masking. They employed SVM as the classifier using three different types of kernels: linear kernel, polynomial kernel, and radial basis function (RBF). Based on the results of the performance comparison, the RBF appears to perform best among the three kernels, with an accuracy of 95.2%, specificity of 90.5%, and sensitivity of 99.8%.

In another approach, Song et al. [35] proposed an improved semi-supervised learning method for extracting additional information from unlabelled cataract fundus images to improve the accuracy of the basic model trained exclusively on marker images. Semi-supervised learning can improve performance by training the classifier with both labelled and unlabelled data. In addition, it can be used to improve supervised classifiers by utilising additional unlabelled data that are typically easier to obtain. The authors previously used the tri-train method, which is also a supervised method, to classify and grade cataracts [36]. The extracted wavelet and texture features were used to train two fundamental models: a Bayesian network and a decision tree. Subsequently, the histogram equalization method was applied to enhance the fundus image during pre-processing. The authors then extracted three features from the enhanced fundus images: texture, wavelet, and sketches, which they used to train a semi-supervised model. They concentrated on methods for updating instance weights and combining multiple binary classifiers into a single powerful multi-classifier, and finally chose logistic regression (LR) and support vector machine (SVM) as baseline models for comparison. Their work achieved an accuracy of 88.6% on the SVM model for the four-category experiment. This was significantly better than their previous work, which only achieved 86% accuracy.

In addition to these, several approaches have been developed for automated cataract detection using slit-lamp images. Since nuclear cataracts affect the nucleus of the ocular lens, automatic cataract detection and grading are performed by extracting features from the nucleus region [22]. H. Li et al. [37], for example, investigated an algorithm for the automatic diagnosis of nuclear cataracts. The anatomical structure of the lens was determined from the captured images using a modified active shape model (ASM), with local features extracted in accordance with the clinical grading protocol. The grades were predicted using support vector machine regression. For the first time, the nucleus region was detected automatically from slit-lamp images, which is critical for assessing nuclear cataracts. Moreover, the proposed improvements to the modified ASM to fit the shape model more robustly to a new image. They achieved a 95% success rate for structure detection and an average grading difference of 0.36 on a 5.0 scale, indicating a promising start towards improving grading objectivity and potentially reducing the workload of ophthalmologists.

In another instance, Huang et al. [38] developed a novel computer-aided diagnosis method based on ranking to facilitate nuclear cataract grading in accordance with established clinical decision-making processes. They predicted the grade of nuclear cataracts from slit-lamp images by comparing them to neighbouring labelled images in a ranked image list generated by a learned ranking function. They viewed the nuclear cataract grading task in their study as a ranking process guided by intuition, and ranking can produce a better fit for the task. In addition, they proposed a new method for “learning to rank” based on the listwise approach, which entails direct optimization for learning ranking functions within their “grading by ranking” scheme. They achieved a 95% grading accuracy with their proposed method, which is higher than the other two existing nuclear cataract grading methods, “grading via classification” [39] and “grading via regression” [40], which achieved 76.8% and 87.3% accuracy, respectively.

In a separate report, Jagadale & Jadhav [41] proposed a simpler automatic system for nuclear cataract classification based on a pupil detection region algorithm that takes advantage of regional properties. They observed a difference in the intensity values of the pupil and the iris in their study, with the pupil in the eye without cataracts registering a darker shade and the iris registering a lighter shade. The shades are reversed for both the pupil and the iris in a cataract-affected eye. As a result, they separated the pupil and the iris using an intensity gradient. They demonstrated a method for cataract detection by extracting the best features from the pupil detection method using the circular Hough Transform (CHT) and correlating them to regional properties. A.B. Jagadale, Sonavane, & Jadav [42] also proposed a computer-aided system for the early detection of nuclear cataracts using CHT in another example. The proposed steps include lens localization using CHT, segmentation of the lens, feature extraction (i.e., mean, correlation, energy, homogeneity, and contrast), and categorization using a multidimensional SVM. Their proposed system detected nuclear cataracts with an accuracy of 90.25%. The results demonstrated a commendable effort to minimize intra- and intergradation variation in comparison to the subjective method currently used by ophthalmologists.

Table 1 summarizes the proposed methods for cataract detection and grading using machine learning and image processing. It can be observed from the Table that most of the proposed methods achieved high accuracy for cataract detection and grading using fundus and slit-lamp images as image modalities. Some of the methods still require human intervention [37,41,42], and most of the research has focused on the detection and grading of nuclear cataracts. There are still limited works on other types of cataracts, such as cortical and posterior subcapsular cataracts. Furthermore, the methods are either semiautomated or not fully automated. In the future, it is anticipated that more robust and fully automated methods will be developed for cataract detection and grading.

### 3.2. Exploitation of Deep Learning Approaches for Cataract Detection

There are also various approaches that make use of deep learning techniques. For instance, Zhang et al. [43] proposed an automatic cataract detection and classification method by visualizing several feature maps at the pool5 layer with their high-order empirical semantic meaning, which provides an explanation for the feature representation extracted by a deep convolutional neural network (DCNN). They eliminate uneven illumination during pre-processing by converting RGB colour images to green channel images. They used DCNN with eight layers for classification and grading, with the first five being convolutional layers and the remaining three being fully connected layers. The output of the final fully connected layer is fed into a four-way SoftMax, which generates a distribution over the four class labels. They conducted two experiments to determine the effects of G-filters on eliminating uneven illumination in fundus images and the effect of database scalability on DCNN classification accuracy. The first experiment demonstrated that the accuracy of the database containing G channel images is significantly higher than that of the database containing RGB colour images, which is 93.52% and 89.92%, respectively. A more stable result for classification accuracies can be obtained by increasing the amount of data. This implies that as the amount of data increases, the classification accuracy of DCNN increases.

In another instance, Zhou, Li, and Li [44] proposed a novel method for automatic cataract classification using fundus images and a deep neural network with discrete state transition (DST). They proposed DST and exponential DST (EDST) as techniques for avoiding overfitting and minimizing storage memory requirements during network training and implementation. This contribution advances the state of the art in cataract grading accuracy. They use a multilayer perceptron (MLP) with exponential discrete parameters, weights, and activations in the input, hidden, and output layers that are constrained in an exponential or uniform discrete space for the classifier. As a result, they achieved a detection accuracy of 94% for DST-ResNet and a grading accuracy of 78.57%, which is the highest among published works using ResNet. Moreover, they were able to avoid overfitting and reduce the memory requirements of their hardware by implementing DST and EDST on a small training set. In a separate example, cataract detection using the CNN with the VGG-19 model was proposed by Mahmud Khan et al. [45]. The pre-processing step involves image cropping to a size of 224 × 224 pixels for all fundus images. Despite using fundus images with unfiltered and unassessed image quality, they managed to achieve an accuracy of 97.47% for the training.

In another work, Xiong et al. [46] proposed a method to classify cataracts by extracting high-level features from a pre-trained residual network (ResNet) adapted from the residual learning framework [47]. To expand the dimension, the high-level features will be fused with texture features extracted from the Gray-level Co-occurrence Matrices (GLCM). The fused feature vectors will then be used to train and verify the 6-class cataract classification using a support vector machine (SVM). The addition of texture features enables the retention of a large amount of information in the original image, allowing for the highest possible accuracy in feature fusion validation. After obtaining the optimal hyperplane through parameter adjustment, they achieved an accuracy of 91.5% with the proposed method, compared to 90.2% with the Softmax classifier.

Li et al. [48] proposed a novel concept of interpretable learning to explain the results of CNN-generated cataract detection. Their contributions include reorganizing AlexNet and GoogLeNet into AlexNet-CAM and GoogLeNet-CAM, respectively, by substituting a global average pooling layer with two fully connected layers. Additionally, they employed Grad-CAM, an enhanced technology based on CAM (class activation mapping), which, combined with visualization, generates a heatmap highlighting significant pathological features. They achieved high accuracies of 93.28% and 94.93% for AlexNet-CAM and GoogLeNet-CAM, respectively. AlexNet-CAM outperforms AlexNet by 1.2%, while GoogLeNet-CAM outperforms GoogLeNet by 0.45%. Nonetheless, all four models are highly accurate at classifying cataracts, and the restructuring of both methods using CAM demonstrates that high accuracy can be maintained. Furthermore, the heatmap generated by Grad-CAM can show the entire lens with numerous large and small vessels highlighted, which can assist ophthalmologists in interpreting the results following cataract detection.

Imran et al. [49] worked towards an automated identification of cataract severity by proposing a hybrid model that integrates a deep learning model and SVM for 4-class cataract classification. They employed transfer learning-based models, which are AlexNet, ResNet, and VGGNet, for automatic feature extraction and SVM, which performs as a recogniser. They managed to achieve 95.65% accuracy with their proposed methods. In another example, Imran et al. [50] also proposed a hybrid convolutional and recurrent neural network (CRNN) for cataract classification. They adopted transfer learning models (AlexNet, GoogLeNet, ResNet, and VGGNet) for multilevel feature representation extraction and analysed the performances of the models on cataract classification. They managed to achieve a high accuracy of 97.39% for the 4-class cataract classification with their proposed method.

The report by Gao, Lin, & Wong [51] serves as an example of studies that utilise deep learning of slit-lamp images. The authors proposed an automated system for grading the severity of nuclear cataracts using slit-lamp images. Nuclear cataracts are typically identified by a uniform increase in the opacification and colouration of the lens nucleus, which can be seen clearly in slit-lamp cross-sectional views of the lens. In their paper, they used unsupervised convolutional-recursive neural networks (CRNNs) for feature learning. They first detected the lens structure and segmented the anatomical sections of the lens. They applied the CRNN to each section to learn a representation for that part of the lens, and for the final step, they applied support vector regression (SVR) to the concatenated features to estimate the cataract grade. Their proposed system achieved an exact agreement ratio of 70.7% when compared to clinical integral grading, an error rate of 88.4% for decimal grading, and a mean absolute error of 0.304.

In a separate study, Qian, Patton, Swaney, Xing, & Zeng [52] classified different areas of cataracts in the lens using supervised training of convolutional neural networks. The proposed steps include image pre-processing, data balancing, data expansion, and the construction of a training model. For the training model, they applied transfer learning and SqueezeNet in their model to save time on training and changing the model. Squeezenet is one type of CNN model that requires fewer parameters than AlexNet but achieves the same level of accuracy. They have managed to achieve a validation accuracy of 96.1% for their proposed method.

In another example, a novel deep learning method was proposed by Zhang et al. [53] to classify nuclear cataracts based on anterior segment OCT images using a convolutional neural network (CNN) model named GraNet. They used a grading block for high-level feature learning that was based on the pointwise convolutional method. They also used a simple cross-training method to further improve the classification performance. The results reported an accuracy of less than 60% for all CNN models, including the proposed model. This was explained by the fact that CNN models use the entire lens structure as input, which also contains the crystalline lens opacities of other types of cataracts and can make it difficult for CNN models to accurately distinguish between different levels of nuclear cataracts.

Table 2 shows the summary of the proposed methods that applied deep learning to various image modalities. From the table, it can be concluded that most of the researchers achieved high accuracy with their proposed methods that applied deep learning in cataract detection and grading. However, for deep learning models, a large dataset is needed for training to achieve better classification or grading [43,49,50]. In addition, the proposed methods still lack a fully automated cataract detection and grading system. Some of the methods could be applied to only one type of cataract. The grading systems of mixed types of cataracts must be developed in the future.

### 3.3. Available Tools for Cataract Grading

OCT is a non-invasive ocular imaging modality that utilises near-infrared light to generate high-resolution images of the flesh microstructure [14]. Anterior segment OCT has been used to grade cataracts by assessing a variety of different features. For example, Kim, Park, and Tchah [54] used AS-OCT with a liquid optics interface to perform a quantitative analysis of whole lens and nuclear lens densities. Additionally, they compared their results to the LOCS III lens grading and corrected distance visual acuity (BCVA). They discovered that nuclear density had a stronger positive correlation with LOCS III than whole lens density. This indicated that the nuclear opalescence of the LOCS III grading system can be classified as the colour of the cataract nucleus. However, the higher correlation between lens nuclear density and LOCS III score compared to whole lens density could be caused by the difficulty identifying the margins encompassing the entire lens capsule because the posterior shadowing of pupils concealed the margin of the lens cortex when pupils were fully dilated. Apart from that, this evaluation is limited to age-related cataracts, excluding all other types. The small number of cases also serves as a constraint on the study’s ability to verify repeatability and reproducibility.

Panthier et al. [55] proposed an objective method for cataract grading based on the quantification of average lens density using SS-OCT scans. They discovered that, by setting the cut-off value for the average lens densitometry (ALD) index to 73.8-pixel units, their method achieved 96.2% sensitivity and 91.3% specificity in detecting cataracts. They previously used an ALD cut-off threshold of 82.9 pixel units [56] but achieved only 73.9% sensitivity and 91.2% specificity (96.2% sensitivity and 91.3% specificity). They increased sensitivity significantly by analysing multiple B-scans passing through different axes for a global 3-dimensional lens analysis, resulting in reliable and reproducible results.

In another work, Chen et al. [57] conducted a lens nuclear opacity quantification study using long-range SS-OCT and evaluated the correlation of their method with the LOCS III and Scheimpflug imaging-based grading systems (Pentacam Nuclear Stage function; PNS). They concentrated on the cataractous nucleus-induced backscatter intensity generated by the long-range SS-OCT images, which were then processed and analysed using ImageJ software to determine the feasibility and advantage of this technique for characterizing the degree of nuclear opacity. They were able to establish strong correlations between the SS-OCT nuclear density and the LOCS III and PNS functions.

The available tools for cataract grading are summarized in Table 3. These methods used AS-OCT and SS-OCT images to evaluate lens density and opacity, and they showed a good correlation with the current traditional grading system LOCS III. However, all the methods are only semiautomated, and some of them only focus on nuclear cataracts. Therefore, a fully automated cataract grading system that also works with other types of cataracts and correlates well with LOCS III or Wisconsin is still needed.

## 4. Modern Trends in Cataract Screening

Today, digital images from digital cameras and smartphones are more widely used for the development of health-related apps in the healthcare sector. Globally, most individuals own a smartphone with easy access to a camera that provides good image quality. Other imaging modalities, such as slit-lamp and fundus images, usually require equipment that is not portable, and the operations usually require skilled professionals. The advance of digital imaging in medical science has greatly helped artificial intelligence (AI) in pattern recognition using CAD systems. CAD systems are intended to assist physicians by automatically interpreting images, which results in decreases in human dependency, boosts the rate of diagnosis, and lowers total treatment costs by reducing false-positive and false-negative (FN) predictions [58]. In addition, anterior segment photographed images that focus on the anterior part of the eyes have also been used for ocular disease detection [59,60]. For that reason, some researchers have started to explore the use of digital camera images from smartphones for early cataract detection and screening.

For example, Fuadah, Setiawan, Mengko, & Budiman, [61] investigated the optimal combination of statistical texture features in digital images that provides the highest level of accuracy for cataract detection. They used K-nearest neighbour (k-NN) classification as the classification method, which will be implemented on the Android smartphone interface. They classified statistical texture analysis into two types for feature extraction: first-order and second-order statistical texture methods. They distinguished between cataract and normal images using the Gray Level Co-occurrence Matrix (GLCM). Then, they calculated the candidate texture measurements for the acquired co-occurrence matrix, such as contrast, dissimilarity, uniformity, correlation, and homogeneity. They discovered that texture feature correlation and homogeneity had no effect on accuracy, implying that the only relevant features are uniformity, contrast, and dissimilarity. They achieved the highest accuracy of 97.5% with a k-value of one for the classification result.

Agarwal, Gupta, Vashisht, Sharma, & Sharma [62] also proposed smartphone-based Android applications that were developed using the proposed methodology and can be used for cataract detection. They utilised the combination of machine learning and image processing techniques in their study to develop the proposed mobile applications. They used k-NN for the classification to reduce the computation time while the mobile applications were under development. They also compared their proposed model with other models, such as SVM and naïve Bayes. According to their results, the proposed model showed higher scores in accuracy (83.07%), F-score (82.97%), recall (82.7%), and precision (83.18%) than the other two models.

Apart from the k-NN model, Sigit, Triyana, & Rochmad [63] proposed a smartphone application for cataract detection that used a single layer perceptron method to distinguish between normal eyes, immature cataract eyes, and mature cataract eyes. They segmented the ocular pupil region using Canny Edge Detection and the Hough Circle Transform and extracted features such as the mean intensity value and uniformity value in the pupil. They achieved a classification accuracy of 100% for normal eyes, 85.7% for eyes with immature cataracts, and 60% for eyes with mature cataracts.

Recently, some works have been utilising smartphones as tools for cataract screening. Ik et al. [64] introduced a mobile cataract screening using a smartphone that uses a red reflex method. Their method focuses on the self-screening cataract mobile application that enables the public to carry out the early detection from the smartphone with camera and flash. Their initial results showed that they still need to do more research on the flash timing, the duration needed for the human eye to be in the dark to capture a clear red reflex, the intensity of the room lighting, and the effects of vertical angle towards the clarity of the red reflex. In another example, da Cunha et al. [65] have proposed an embedded teleophthalmology system that uses a smartphone called TriOft for the screening of cataract. The system is based on image processing and expert systems that consist of an off-line mobile pre-diagnostic platform for cataract screening in remote areas. Their proposed system managed to achieve 90% accuracy which is higher than the accuracy obtained by ophthalmologists (62.5%) and slightly lower than the accuracy from the OPTICA system (95.31%).

**Figure 3:**
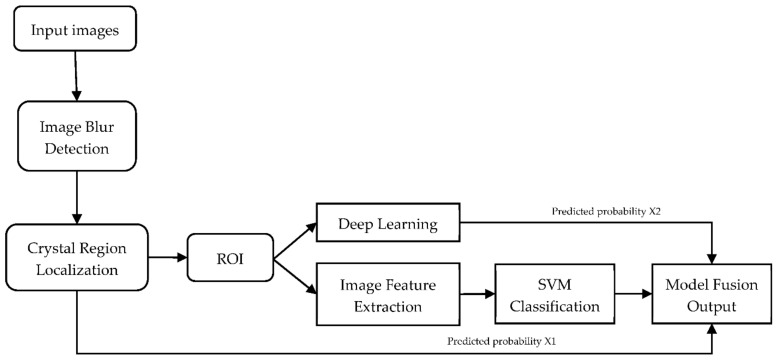
Unified framework for automated nuclear cataract severity classification [66].

Besides, there have also been a few works that utilise smartphones attached with slit-lamp adapters for cataract screening purposes. For example, Hu et al. [66] proposed a unified framework for automated nuclear cataract severity classification using smartphone-based slit-lamp images. Their framework as shown in Figure 3 [66] integrates both deep learning and traditional feature extraction methods. They employed YOLOv3 to locate the nuclear region of the ocular lens image. Then, they intercepted the nuclear region of the original image to obtain a nuclear region dataset and used the ShuffleNet and SVM classifiers for cataract grading. Comparing their proposed algorithm with the GoogleNet and ResNet-101 methods, their proposed algorithm managed to produce the higher value for accuracy (93.48%), sensitivity (89.2%), Youden (0.846), F1 (92.3%), and Kappa (0.954). In another work, Yazu et al. [67] evaluated nuclear cataract detection using a smartphone-attachable slit-lamp device called Smart Eye Camera (SEC) and a conventional slit-lamp microscope. During the evaluation, the pupil of the subjects was dilated and examined using both approaches. Their results showed that the nuclear cataract grading by both approaches showed a significant correlation, which suggests that the SEC approach is as reliable as the conventional slit-lamp microscope approach for evaluating cataracts.

## 5. Challenges and Future Direction

Cataract detection and grading methods developed in the past few years indicate that more robust and fully automated cataract detection and grading systems are still needed. Imaging modalities such as fundus, slit-lamp, and OCT images require medical equipment that is expensive and not portable. Some of the previously developed methods have limitations that need to be overcome. For example, although many automated image-based cataract grading methods have been proposed, they are limited to nuclear cataract and use the slit-lamp photo only. Such grading methods of subcapsular and cortical cataracts are still unavailable because the latter cases’ cloud formation is more difficult to determine the maturity state. Currently, there have been a few studies on portable smartphone-based slit-lamp images for cataract screening. However, those methods involve pupil dilation, which is an invasive procedure needed during the evaluation. Since digital images from smartphones are cheaper, more portable, and adaptable, they can be a practical solution for automated cataract detection and grading. In addition, it can also help the ophthalmologist particularly in rural areas with limited access to quality healthcare facilities. For this reason, cataract screening using mobile devices such as smartphones could be exploited as an alternative solution.

In recent years, the term connected health has gained popularity to describe the new technology-enabled model of healthcare delivery. According to Caulfield & Donnelly [68], connected health can be defined to encompass categories such as wireless, digital, electronic, mobile, and tele-health. It also refers to the conceptual model for health management, where devices, services, or interventions are designed based on the patient’s need for the sharing of health-related data so that the patient can receive care in the most interactive and efficient way. Moreover, the significant application of information and communication technology in the health sector to date has led to a substantial improvement in the healthcare delivery system. Furthermore, smartphones, which are sophisticated devices that combine traditional mobile phone features with advanced computing capabilities that allow users to access software programs, have gained immense popularity for health-related purposes. The smartphone’s technological capability, popularity, availability, and globally increased ownership have helped encourage the smartphone as an appealing tool for patient self-management, continuous symptom and vital sign monitoring, and patient-physician communication [69,70].

**Figure 4:**
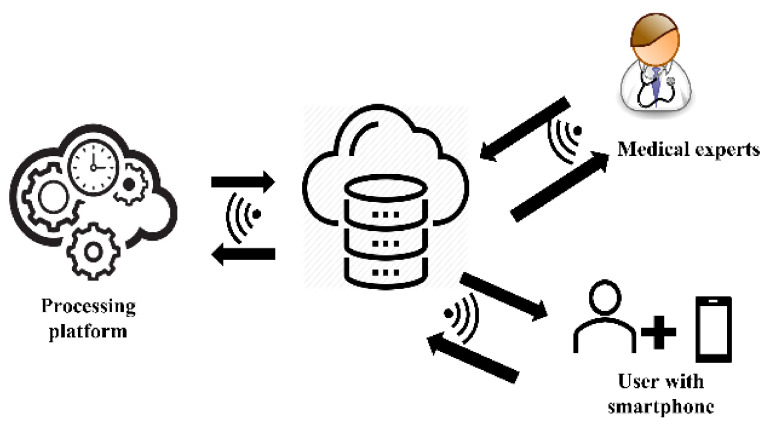
Framework for a connected cataract screening system using smartphone.

In addition, AI has significant applicability in healthcare since it can handle and utilise very complicated datasets that exist in very complex systems [71]. In addition, clinical medicine has emerged as an intriguing application area for ML and DL models, where these models already outperforming humans in clinical pathology, radiography, ophthalmology, and dermatology [72]. The rapid growth of both technologies can be further explored as a potential framework for portable cataract screening tools that can be used to assist ocular healthcare practitioners, particularly in remote areas with limited access to quality healthcare facilities. Figure 4 illustrates the framework of a connected cataract screening system that uses anterior segment photographed images (ASPIs), which are digital eye images captured with a smartphone camera. The user may capture ASPIs using the smartphone camera, and the images are uploaded to the cloud data storage. The images are then analyzed on the cloud computing platform using a suitable machine learning algorithm to detect and grade cataracts. Finally, the result can be notified to the user via a unified messaging system.

## 6. Conclusions

Significant progress has been made over the years in developing automated cataract detection and grading systems that utilise four distinct imaging modalities: OCT images, fundus images, slit-lamp images, and digital camera images. These efforts have demonstrated that they can help relieve ophthalmologists’ burdens associated with cataract diagnosis, as the proposed methods are less time-consuming, and most of the methods achieve high accuracy. However, some gaps remain to be filled in the future, including the development of more robust, portable, and fully automated cataract detection and grading systems. Based on the information presented in this review, a highly promising approach for portable connected cataract screening using smartphones, along with the application of machine learning and deep learning, can be further explored in the future. This approach could be highly beneficial for ocular healthcare practitioners, especially in rural areas where access to quality healthcare facilities is limited.

## Figures and Tables

**Table 1:** Summary of Previous Methods Using Machine Learning and Image Processing.

Authors	Methods	Image Modality	Achievement	Limitation	Database
Yang et al. [33]	Automatic cataract classification with an improved version of top-bottom hat transformation as part of their pre-processing and 2-layer backpropagation (BP) neural network as classifier	Fundus	Achieve true positive rate of 82.1% (training) and 82.9% (test)	Pre-processing takes longer for a single image	Beijing TONGREN Hospital (504 fundus images)
Behera et al. [34]	Nuclear cataract detection based on image processing and machine learning	Fundus	Achieve overall accuracy of 95.2%	Focused only on nuclear cataract	Kaggle and GitHub repository (800 fundus images)
Song et al. [35]	Proposed an improved semi-supervised learning method to acquire some additional information from unlabelled cataract fundus images to improve the accuracy of the basic model to train only the marker images	Fundus	Achieve accuracy of 88.6% using SVM model	Semiautomated method Require labelled data	7851 fundus images
H. Li et al. [37]	The anatomical structure of the lens images is detected using a modified active shape model (ASM) where the local features are extracted according to the clinical grading protocol and utilises a support vector machine regression for the grade prediction	Slit-lamp	Achieve a 95% success rate for structure detection and an average grading difference of 0.36 on a 5.0 scale	User intervention was provided for the images with inaccurate focus, small pupil, or dropping eyelid	Singapore Malay eye study (SiMES) (5850 slit-lamp images)
Huang et al. [38]	Novel computer-aided diagnosis method by ranking to facilitate nuclear cataract grading that followed conventional clinical decision-making process.	Slit-lamp	Achieve a 95% grading accuracy compared to other methods “grading via classification” (76.8%) and “grading via regression” (87.3%)	Focused only on nuclear cataract	Singapore Malay Eye Study (SiMES) (1000 slit-lamp images)
Amol B. Jagadale & Jadhav [41]	Simpler automatic systems for nuclear cataract classification from the development of pupil detection region algorithm using region properties	Slit-lamp	Proposed best features from pupil detection method using circular Hough Transform (CHT)	Need human intervention A simple method to classify only nuclear cataract cases	Cottage Hospital, Pandharpur and Lions eye Hospital, Miraj
A.B. Jagadale et al. [42]	Proposed an early detection of nuclear cataract	Slit-lamp	Achieved 90.25% accuracy in detecting nuclear cataract	Need human intervention The proposed method showed a low performance for specificity with only 63.4%	Government hospital Pandharpur (2650 slit-lamp images)

**Table 2:** Summary of Cataract Detection Using Deep Learning Approaches.

Authors	Methods	Image Modalities	Achievement	Limitation	Database
Zhang et al. [43]	Visualize some of the feature maps at pool5 layer with their high-order empirical semantic meaning that provides an explanation to the feature representation extracted by deep convolutional neural network (DCNN)	Fundus	Achieve accuracy of 93.52% (detection) and 86.69% (grading)	Accuracy can be increased by increasing the amount of data, therefore, big data is needed	Beijing Tongren Eye Center of Beijing Tongren Hospital (5620 fundus images)
Zhou, Li, and Li [44]	Deep neural network with discrete state transition (DST)	Fundus	Achieve 78.57% for cataract grading (with prior knowledge)	Lower accuracy compared to previous DST-ResNet for cataract grading (without prior knowledge) Automated method and does not need prior knowledge	Beijing Tongren hospital (1355 fundus images)
Mahmud Khan et al. [45]	Cataract detection using the CNN with VGG-19 model	Fundus	Achieve high accuracy of 97.47%	Use unfiltered and quality unassessed fundus images	Shanggong Medical Technology Co., Ltd. (800 fundus images)
Xiong et al. [46]	Grade cataracts using a pre-trained residual network (ResNet) which is adapted from residual learning framework [47] to extract high-level features	Fundus	Achieve 91.5% accuracy for 6 class classification	Good results in classifications 0 and 5 but does not effectively distinguish between 2 and the adjacent classifications	1352 fundus images
Li et al. [48]	Restructured AlexNet and GoogleNet into AlexNet-CAM and GoogleNet-CAM, respectively and use Grad-CAM which is an improved technology on basis of Class Activation Mapping (CAM)	Fundus	Achieve accuracy of 93.28% (AlexNet-CAM) and 94.93% (GoogLeNet-CAM)	Automated method Require labelled data	Beijing Tongren Eye Center of Beijing Tongren hospital (5620 fundus images)
Imran et al. [49]	Hybrid model that integrates deep learning model and SVM for 4-class cataract classification	Fundus	Achieve 95.65% accuracy	Limited fundus images for moderate and severe cataract categories	Tongren Hospital, China (8030 fundus images)
Imran et al. [50]	Hybrid convolutional and recurrent neural network (CRNN) for the cataract classification	Fundus	Achieve accuracy of 97.39% for 4-class cataract classification	Limited fundus images for moderate and severe cataract categories	Tongren Hospital, China (8030 fundus images)
Gao, Lin, & Wong [51]	Automatically learn features for grading the severity of nuclear cataracts from slit-lamp images using unsupervised convolutional-recursive neural networks (CRNN) method	Slit-lamp	Achieve 70.7% exact agreement ratio against clinical integral grading, 88.4% decimal grading error ≤ 0.5, 99.0% integral grading error ≤ 1.0 and MAE of 0.304	The results might be affected by the error in the human-labelled ground truth	ACHIKO-NC Dataset (5378 images)
Qian, Patton, Swaney, Xing, & Zeng [52]	Utilise supervised training of convolutional neural network to classify different areas of cataracts in lens	Slit-lamp	Achieve validation accuracy of 96.1%	Need human intervention High value of validation loss	No. 2 Hospital, Changshu, Jiangsu, China (420 slit-lamp images)
Zhang et al. [53]	Nuclear cataract classification based on the anterior segment OCT images using Convolutional Neural Network (CNN) model named GraNet	OCT	Achieve accuracy of less than 60% for all CNN models	Imbalanced dataset 2D AS-OCT images might not contain enough pathology information of cataract	Dataset acquired by CASIA2 device of Tomey Corporation, Japan (38,225 OCT images)

**Table 3:** Available Tools for Cataract Grading.

Authors	Methods and Tools	Achievement	Limitation	Database
Kim et al. [54]	Evaluated correlation of LOCS III lens grading with nuclear lens density and whole lens density using AS-OCT with liquid optics interface	Nuclear density showed a higher positive correlation with LOCS III compared to the whole density	Need human intervention Limited number of datasets Only studied the dense nuclear cataracts	Asan Medical Center
Panthier et al. [55]	Cataract grading method based on average lens density quantification with SS-OCT scans	Achieve d96.2% (sensitivity) and 91.3% (specificity)	A single-centre study that delineated the anterior and posterior cortex Do reproduce for reliable score for subgroup analysis	Rothschild Foundation, Paris, France
Chen et al. [57]	Evaluated the correlation of lens nuclear opacity quantitation by long-range SS-OCT method with LOCS III and Scheimpflug imaging-based grading system	Obtained a good correlation between SS-OCT nuclear density and LOCS III and Pentacam nuclear density	Semiautomatic and time-consuming Only studied nuclear cataracts	Uses 120 images
